# Allogeneic hematopoietic stem cell transplantation for NK/T-cell lymphoma: an international collaborative analysis

**DOI:** 10.1038/s41375-023-01924-x

**Published:** 2023-05-08

**Authors:** Philipp Berning, Norbert Schmitz, Maud Ngoya, Hervé Finel, Ariane Boumendil, Fengrong Wang, Xiao-Jun Huang, Olivier Hermine, Laure Philippe, Lucile Couronné, Arnaud Jaccard, Daihong Liu, Depei Wu, Hans Christian Reinhardt, Yves Chalandon, Eva Wagner-Drouet, Mi Kwon, Xi Zhang, Ben Carpenter, Ibrahim Yakoub-Agha, Gerald Wulf, Javier López-Jiménez, Jaime Sanz, Hélène Labussière-Wallet, Avichai Shimoni, Peter Dreger, Anna Sureda, Won Seog Kim, Bertram Glass

**Affiliations:** 1 Department of Hematology and Oncology, University Hospital Muenster, Muenster, Germany; 2 European Society for Blood and Marrow Transplantation, Hôpital St. Antoine, Paris, France; 3 Beijing University, Institute of Hematology, National Clinical Research Center for Hematologic Disease, Beijing, China; 4 Department of Hematology, Hôpital Necker, Assistance Publique Hôpitaux de Paris, University Paris Descartes, Paris, France; 5 Laboratory of Onco-Hematology, Necker Children’s Hospital, Assistance Publique-Hôpitaux de Paris (APHP), Université Paris Cité, Paris, France; 6 Department of Hematology, CHU Dupuytren, Limoges, France; 7 General Hospital of People’s Liberation Army of China, Beijing, China; 8 National Clinical Research Center for Hematologic Diseases, Jiangsu Institute of Hematology, The First Affiliated Hospital of Soochow University, Soochow, China; 9 Department of Hematology and Stem Cell Transplantation, University Hospital Essen, University Duisburg-Essen, Essen, Germany; 10 Division of Hematology, Hôpitaux Universitaires de Genève and Faculty of Medicine, University of Geneva, Geneva, Switzerland; 11 Department of Medicine III, Johannes Gutenberg-University Mainz, Mainz, Germany; 12 Department of Hematology, Gregorio Marañón General University Hospital, Institute of Health Research Gregorio Marañón, Madrid, Spain; 13 Medical Center of Hematology, Xinqiao Hospital, Army Medical University, Chongqing, China; 14 Department of Haematology, University College London Hospitals, London, UK; 15 CHU de Lille, INSERM U1286, Infinite, Université de Lille, Lille, France; 16 Department of Hematology/Oncology, Georg-August-University Göttingen, Göttingen, Germany; 17 Hematology Department, Hospital Universitario Ramón y Cajal, Madrid, Spain; 18 Department of Hematology, Hospital Universitario y Politécnico La Fe, Valencia, Spain; 19 Centre Hospitalier Lyon Sud, Pavillon Marcel Bérard, Service Hematologie, Lyon, France; 20 Division of Hematology and Bone Marrow Transplantation, Chaim Sheba Medical Center, Tel-Hashomer, Israel; 21 Department of Medicine V, University of Heidelberg, Heidelberg, Germany; 22 Hematology Department, Institut Català d’Oncologia Hospitalet, IDIBELL, Universitat de Barcelona, Barcelona, Spain; 23 Samsung Medical Center, Sungkyunkwan University School of Medicine, Seoul, Korea; 24 Department of Hematology and Stem Cell Transplantation, Helios Clinic, Berlin-Buch, Germany

**Keywords:** T-cell lymphoma, T-cell lymphoma

## Abstract

Natural killer/T-cell lymphomas (NKTCL) represent rare and aggressive lymphoid malignancies. Patients (pts) with relapsed/refractory disease after Asparaginase (ASPA)-based chemotherapy have a dismal prognosis. To better define the role of allogeneic hematopoietic stem cell transplantation (allo-HSCT), we conducted a retrospective analysis of data shared with the European Society for Blood and Marrow Transplantation (EBMT) and cooperating Asian centers. We identified 135 pts who received allo-HSCT between 2010 and 2020. Median age was 43.4 years at allo-HSCT, 68.1% were male. Ninety-seven pts (71.9 %) were European, 38 pts (28.1%) Asian. High Prognostic Index for NKTCL (PINK) scores were reported for 44.4%; 76.3% had >1 treatment, 20.7% previous auto-HSCT, and 74.1% ASPA-containing regimens prior to allo-HSCT. Most (79.3%) pts were transplanted in CR/PR. With a median follow-up of 4.8 years, 3-year progression-free(PFS) and overall survival were 48.6% (95%-CI:39.5–57%) and 55.6% (95%-CI:46.5–63.8%). Non-relapse mortality at 1 year was 14.8% (95%-CI:9.3–21.5%) and 1-year relapse incidence 29.6% (95%-CI:21.9–37.6%). In multivariate analyses, shorter time interval (0–12 months) between diagnosis and allo-HSCT [HR = 2.12 (95%-CI:1.03–4.34); *P* = 0.04] and transplantation not in CR/PR [HR = 2.20 (95%-CI:0.98–4.95); *P* = 0.056] reduced PFS. Programmed cell death protein 1(PD-1/PD-L1) treatment before HSCT neither increased GVHD nor impacted survival. We demonstrate that allo-HSCT can achieve long-term survival in approximately half of pts allografted for NKTCL.

## Introduction

NK/T-cell lymphomas (NKTCL) represent a rare and aggressive entity belonging to the group of mature T-cell and NK-cell neoplasms [1]. NKTCL are relatively frequent in patients of Asian and South American origin accounting for 5–10% of all lymphomas in these regions while being less widespread in Europe and the United States [1, 2]. Epstein–Barr virus (EBV) infection is universally present in NKTCL and EBV plasma levels constitute a surrogate marker for lymphoma load showing prognostic relevance [3]. Due to the expression of P-glycoprotein in tumor cells leading to a multidrug resistance phenotype, CHOP (cyclophosphamide, doxorubicin, vincristine, prednisolone) and other anthracycline-containing regimens achieve suboptimal treatment results and have largely been abandoned [4, 5]. State-of-the-art regimens include asparaginase (ASPA) often in combination with gemcitabine and platinum or derivates [6–8]. While patients with limited disease (stage I/II) benefit from sequential or concurrent radio-chemotherapy, patients with advanced stages (stage III/IV) are mostly treated with ASPA-containing regimens alone [9, 10]. With such treatment long-term survival is achieved in 40–50% of patients [10, 11]. Reports on autologous transplantation (auto-HSCT) administered for consolidation after first-line treatment gave results comparable to those achieved with ASPA-containing regimens alone suggesting that upfront auto-HSCT does not translate into a survival benefit [12, 13]. Importantly, for patients with refractory or relapsed disease, results of conventional salvage therapy remain poor with median OS and PFS of 6.4 and 4.1 months, respectively [11]. Recently, immune blockade of the Programmed cell death protein 1/Programmed death-ligand 1(PD-1/PD-L1) axis showed promising response rates in such patients; however, robust data on long-term survival currently remain unknown [14, 15]. Allogeneic transplantation of hematopoietic stem cells (allo-HSCT) can provide long-term remissions and cure in selected patients with NKTCL [16, 17]. Accordingly, current guidelines propose allo-HSCT in high-risk patients after ASPA-based chemotherapy [18, 19]. These recommendations, however, are mostly based on smaller retrospective series with less than 20 patients reported decades ago and primarily included Asian patients [16, 17, 20]. To date, the largest series including patients of non-Asian ethnicity was reported by the Center for International Blood and Marrow Transplant Research (CIBMTR) and comprised a total of 82 patients (66 non-Asian patients) with advanced-stage or relapsed/refractory disease [21]. These patients showed 3-year PFS and OS rates of 28% and 34%, respectively. Of note, only 38% of these patients had been treated with ASPA-containing regimens prior to allo-HSCT performed between 2000 and 2014 [21]. Therefore, we sought to investigate disease characteristics, pretreatment, and outcomes of a representative group of patients from Asia and Europe allografted for NKTCL in newer years. Thus, the goal was to provide representative data to better define the role of allo-HSCT in the era of ASPA-containing treatment by analyzing data reported to the European Society for Bone and Marrow Transplantation (EBMT) and cooperating centers in China and South Korea. Furthermore, the role of PD-1/PD-L1 antibodies administered prior to allo-HSCT was investigated.

## Materials and methods

### Data collection

We conducted a retrospective analysis of transplant data registered with the Lymphoma Working Party of the EBMT and the local registries of Samsung Medical Center, Seoul, South Korea; and the First Medical Center of PLA General Hospital, Beijing, China. In total, data were provided by 69 transplant centers in Europe and Asia. For a full list of contributing centers and patient numbers see Supplemental Table 1. Details on the standardized data collection process and quality management can be found elsewhere [22]. All accredited EBMT transplant centers are required to obtain written informed consent prior to data registration with the EBMT following the Helsinki Declaration of 1975. The two cooperating Asian centers signed a project-specific joint controllership agreement confirming that the participating center followed EBMT rules and regulations. We collected data for all consecutive adult patients (≥18 years) diagnosed with NKTCL (aggressive NK-cell leukemias and chronic lymphoproliferative disorders of NK cells excluded) regardless of prior treatment and remission status who received allo-HSCT either as first transplant or after a previous autologous HSCT had failed. Data between 2010 and 2020 were analyzed in order to include patients treated state-of-the-art and to ensure sufficient follow-up time. We included patients allografted with BM and/ or PB from any donor including related, unrelated, or haplo-identical donors. Patients transplanted with cord blood (*n* = 2) were excluded from this analysis. Baseline information, transplantation characteristics as well as outcome data were extracted from the EBMT registry. Identical forms were provided by centers not being EBMT members.

### Definitions

The diagnosis was based on local histological review. Disease stages were classified according to the Ann Arbor system as localized (Stage I/II) and advanced (Stage III/IV); the Prognostic Index for NK/T-cell lymphoma (PINK) scoring system was applied to classify patients belonging to the different risk groups as published previously [23]. Relapse was diagnosed when lymphoma recurred at least 3 months after the end of all therapy in patients having achieved a complete remission. Disease status was assessed by individual investigators according to standard criteria at the time patients were referred for transplantation. Treatment regimens applied before allo-HSCT were classified as Asparaginase-based, anthracycline-based, or gemcitabine-based. Additionally, DeVIC (dexamethasone, etoposide, ifosfamide, carboplatin) and VIPD (etoposide, ifosfamide, cisplatin, dexamethasone) were administered to eight patients.

Regimens containing either TBI with doses >6 Gy, total oral busulfan >8 mg/kg or total intravenous busulfan >6.4 mg/kg body weight were classified as myeloablative conditioning (MAC). All other regimens were classified as reduced-intensity conditioning (RIC) as reported elsewhere [24]. The diagnosis and grading of acute graft-versus-host disease (GVHD) and chronic GVHD were done by transplant centers according to established criteria [25, 26].

### Statistical analysis

The endpoints analyzed were progression-free survival (PFS) defined as survival without lymphoma relapse or progression (patients alive without lymphoma relapse or progression were censored at the time of last contact), overall survival (OS) defined as time from transplantation to death from any cause; non-relapse mortality (NRM) defined as death without previous lymphoma relapse and relapse incidence (RI). All outcomes were measured from the day of transplantation. Surviving patients were censored at the time of the last contact. The probabilities of OS and PFS were calculated using the Kaplan–Meier method. We calculated cumulative incidences for RI and NRM using a competing risk model, where death during remission was treated as a competing event for relapse. Death and relapse were considered as competing events for calculations of aGVHD and cGVHD. Demographics were compared between groups using the chi-squared test or Fisher’s exact test for categorical variables and the Mann–Whitney *U* test for continuous variables. Univariate analyses were performed using the log-rank test for PFS and OS, while Gray’s test was used for CI. Multivariate analyses were performed using the Cox proportional-hazards regression model. Results were shown as hazard ratio (HR) with a 95% confidence interval (95% CI). All tests were two-sided, and the type I error was fixed at 0.05 for factors associated with time-to-event outcomes. All analyses were performed using R statistical software version 4.2.2 (available online at http://www.R-project.org) and IBM SPSS version 26.0 (SPSS Inc., Chicago, IL).

## Results

### Patient characteristics, pretreatment, and transplant modalities

The study population consisted of 135 patients receiving allo-HSCT between 2010 and 2020. Major patient- and procedure-related characteristics are shown in Table 1. With a median age of 43.4 years (range: 18.3–67.7 years) at transplantation and 68.1% of patients being male, the cohort was representative of a typical NKTCL population [27]. Recipients were predominantly European (71.9%), and the other patients were of Asian origin. Advanced disease status at diagnosis (stage III–IV) was observed in 61.4%, and high PINK scores were reported for 44.4% of the patients; 21.5% had low/intermediate PINK scores, while PINK was not available in 34.1% of the cases. First-line therapies are also summarized in Table 1; more than half of the patients (50.4%) received ASPA as part of first-line treatment, 26.7% had received Anthracycline-based regimens, 5.9% were treated with DeVIC or VIPD, one patient with a Gemcitabine-based regimen, for 16.3% other regimen were applied or first-line treatment was unavailable. Seventy-six percent of the patients had received two or more lines of therapy prior to allo-HSCT and 20.7% of the cohort had received auto-HSCT during the treatment course. There were no cases of consolidative allo-HSCT in CR after first-line treatment for patients with localized disease at diagnosis. Of note, prior to allo-HSCT, 74.1% of our patients had received ASPA -containing regimens. PD-1/PD-L1 inhibitors had been administered to 13 patients prior to and in 5 cases after allo-HSCT. Remission status at the time of HSCT was complete remission (CR) in 52.6%, partial remission (PR) in 26.7%, and stable/progressive disease (SD/PD) in 20.0%. Most patients had an unrelated donor or a matched (10/10-matched) related donor (43.7% and 35.6% of the cases, respectively). Of note, 27 patients (20.0%) received a haplo-identical graft; 15 of these patients received post-transplant cyclophosphamide as GVHD prophylaxis, 9 patients received calcineurin inhibitor-based GVHD prophylaxis, while 3 patients had anti-thymocyte globulin-based (*“Beijing protocol”*) GVHD prophylaxis. The stem cell source was peripheral blood for 91.1% of patients. RIC and MAC were used in 46.7% and 53.3% of the cases, respectively. Thirty-five patients (25.9%) had TBI as part of the conditioning 17 of which received a myeloablative TBI dose (>6 Gy). T-cell depletion and GVHD prophylaxis regimens are listed in Table 1.

**Table 1:** Clinical and transplantation characteristics of patients with NKTCL.

Variable	Total cohort *N* = 135 (%)
Male	92 (68.1)
Age at diagnosis, median, range (years)	41.8 (15.8–66.2)
Age at transplantation, median, range (years)	43.4 (18.3–67.7)
Diagnosis—allo-HSCT median, range (months)	11.2 (1.6–257)
Karnofsky performance score before allo-HSCT	
80–100%	85 (63.0)
<80%	6 (4.4)
Unknown	44 (32.6)
Region	
Europe	97 (71.9)
Asia	38 (28.1)
Ann-Arbor stage at diagnosis	
Localized (I-II)	40 (30.0)
Advanced (III-IV)	83 (61.4)
Unknown	12 (8.6)
PINK score	
Low	14 (10.4)
Intermediate	15 (11.1)
High	60 (44.4)
Unknown	46 (34.1)
First-line therapy	
Anthracycline-based	36 (26.7)
Asparaginase-based	68 (50.4)
DeVIC or VIPD	8 (5.9)
Gemcitabine-based	1 (0.7)
Other	14 (10.4)
Unknown	8 (5.9)
Radiotherapy in first-line	44 (32.6)
Prior autologous transplantation	28 (20.7)
Number of prior therapies	
1	27 (20.0)
2	45 (33.3)
3 or more	58 (43.0)
Unknown	5 (3.7)
Asparaginase-containing therapy (any time before HSCT)	100 (74.1)
PD-1/PD-L1 inhibitor treatment	
No PD-1/PD-L1 inhibitor	96 (71.1)
After HSCT	5 (3.7)
Before HSCT	13 (9.6)
Unknown	21 (15.6)
Status at transplantation	
CR	71 (52.6)
PR	36 (26.7)
SD	3 (2.2)
PD	24 (17.8)
Unknown	1 (0.7)
Donor Type	
Matched related donor	48 (35.6)
Unrelated donor	59 (43.7)
Mismatched related donor	28 (20.7)
Haploidentical donor	27 (20.0)
Other	1 (0.7)
Stem cell source	
Bone marrow	12 (8.9)
Peripheral blood	123 (91.1)
Conditioning regimen	
RIC	63 (46.7)
MAC	72 (53.3)
TBI as part of conditioning	35 (25.9)
>6 Gy	17 (12.6)
≤6 Gy	8 (5.9)
TBI, but dose unknown	10 (7.4)
No TBI	100 (74.1)
In vivo T-cell depletion	
ATG	50 (37)
Alemtuzumab	10 (7.4)
No T-cell depletion	75 (55.6)
PTCY	
Yes	35 (25.9)
No	100 (74.1)
GVHD prophylaxis	
Cyclosporin A alone	14 (10.4)
Cyclosporin A + MTX	33 (24.4)
Cyclosporin A + MMF	18 (13.3)
Cyclosporin A + MMF + MTX	27 (20.0)
Tacrolimus + MTX	6 (4.5)
Tacrolimus + MMF	6 (4.5)
Other	30 (22.2)
Unknown	1 (0.7)

*PINK* prognostic index for NK/T-cell lymphoma, *DeVIC* dexamethasone, etoposide, ifosfamide, carboplatin, *VIPD* etoposide, ifosfamide, cisplatin, dexamethasone, *CR* complete response, *PR* partial response, *SD* stable disease, *PD* progressive disease, *RIC* reduced-intensity conditioning, *MAC* myeloablative conditioning, *TBI* total body irradiation, *ATG* anti-thymocyte globulin, *PTCY* post-transplant cyclophosphamide, *GVHD* graft-versus-host disease, *MTX* methotrexate, *MMF* mycophenolate mofetil.

### Transplantation outcomes

**Fig. 1:**
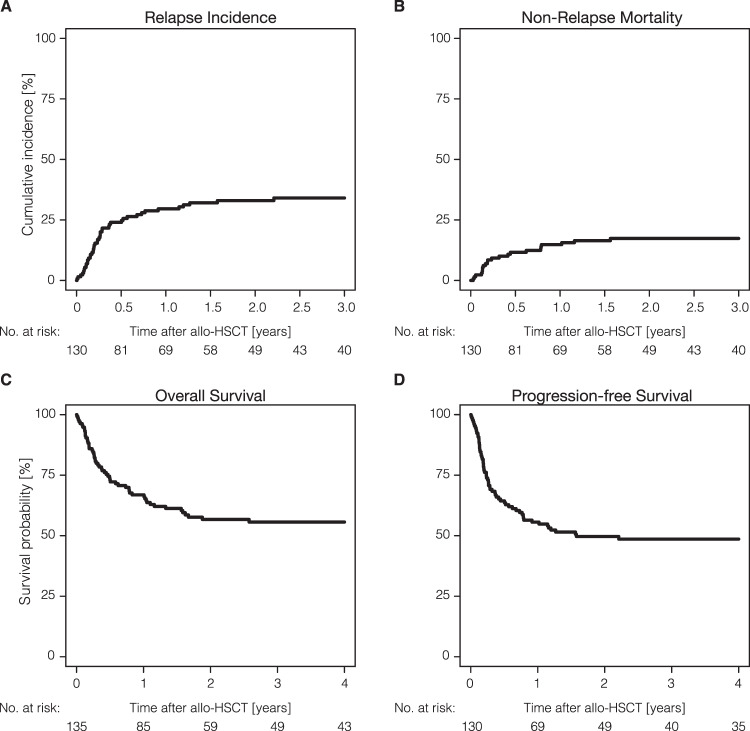
Outcomes of NKTCL patients after allo-HSCT. Key outcome parameters for all NKTCL patients. **A** Cumulative incidence of disease relapse and **B** non-relapse mortality. **C** Kaplan-Meier estimates for overall survival and **D** progression-free survival.

Major outcomes of patients receiving allo-HSCT are shown in Fig. 1 and Table 2. With a median follow-up of 4.8 years (range: 3.5–5.9 years), we noted a relapse incidence (RI) of 29.6% (95% CI: 21.9–37.6%) and 34.1% (95% CI: 25.8–42.4%) at 1 and 3 years; non-relapse mortality (NRM) was 14.8% (95% CI: 9.3–21.5%) and 17.3% (95% CI: 11.3–24.5%) at 1 and 3 years, respectively. The corresponding rates of progression-free survival (PFS) and overall survival (OS) were 55.6% (95% CI: 46.6–63.7%) and 66.9% (95% CI: 58.1–74.2%) at one year, and 48.6% (95% CI: 39.7–57%) and 55.6% (95% CI: 46.5–63.8%) at 3 years, respectively. Of note, no relapse was recorded beyond 3 years after HSCT. The incidence of acute GVHD grades II-IV at day 100 was 18.8% (95% CI: 12.1–26.5%), while the cumulative incidences of chronic GVHD at 1 year and 3 years posttransplant were reported as 29.6% (95% CI: 21.7–37.9%) and 34.5% (95% CI: 25.9–43.1%). Extensive cGVHD was reported as 11.7% (95% CI: 6.5–18.5%) with no new cases beyond the first year after allo-HSCT. Sixty patients had died at last follow-up with disease relapse being the most frequent cause of death in 32 patients (53.3%), followed by HSCT-related causes (including GVHD and infectious complications) in 20 patients (33.3%). Other causes of death were reported in 6 cases (10.0%) including CMV infection, COVID-19-associated ARDS, and a fatal catheter complication in one patient each; infections not directly related to the transplant procedure were reported for the remaining three patients.

**Table 2:** Post-transplantation outcomes for all NKTCL patients.

Outcomes	Evaluable patients (*N*)	Probability (95% CI)
Acute GVHD Grade II-IV	112	
100 days		18.8% (12.1–26.5%)
Chronic GVHD	124	
1 year		29.6% (21.7–37.9%)
2 years		34.5% (25.9–43.1%)
3 years		34.5% (25.9–43.1%)
Relapse incidence	130	
1 year		29.6% (21.9–37.6%)
2 years		33.0% (24.9–41.3%)
3 years		34.1% (25.8–42.4%)
Non-relapse mortality	130	
1 year		14.8% (9.3–21.5%)
2 years		17.3% (11.3–24.5%)
3 years		17.3% (11.3–24.5%)
Progression-free survival	135	
1 year		55.6% (46.6–63.7%)
3 years		48.6% (39.5–57%)
5 years		48.6% (39.5–57%)
Overall survival	135	
1 year		66.9% (58.1–74.2%)
3 years		55.6% (46.5–63.8%)
5 years		52.9% (43.4–61.5%)

*CI* confidence interval, *GVHD* graft-versus-host disease.

### Outcomes across subgroups

**Fig. 2:**
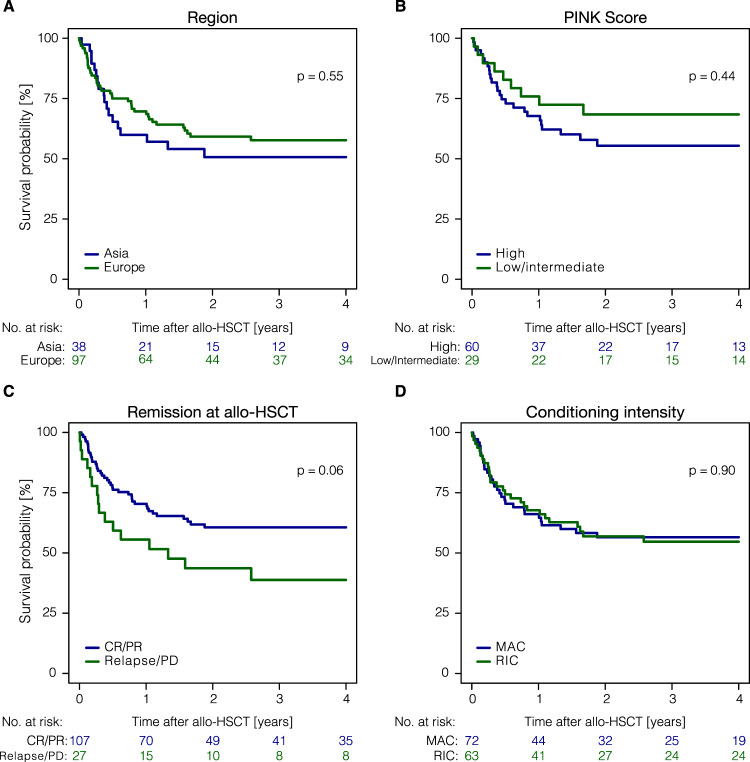
Overall survival of NKTCL patients across selected subgroups. Kaplan-Meier estimates for overall survival of indicated subgroups. **A** Patients transplanted in European or Asian centers. **B** PINK score groups high vs. low/intermediate. **C** Remission status at allo-HSCT shown as complete response (CR)/partial response (PR) vs. relapse/progressive disease (PD). **D** Conditioning intensity is indicated as myeloablative conditioning (MAC) and reduced-intensity conditioning (RIC).

The 1-year and 3-year PFS rates for European and Asian patients were comparable with 1-year PFS rates of 57.8% (95% CI: 47.1–67.1%) and 50.0% (95% CI: 32.9–64.9%) (*P* = 0.56); and 3-year PFS–rates of 50.1% (95% CI: 39.2–60.0%) and 44.4% (95% CI: 28.0–59.6%) (*P* = 0.56) (Table 3). Also, 3-year OS was not significantly different [57.7% (95% CI: 46.8–67.1%) and 50.7% (95% CI: 33.4–65.6%) (*P* = 0.55)] (Table 3, Fig. 2A); OS showed a plateau beyond 3 years indicating that patients surviving 3 years after transplantation may be cured (Fig. 1C). Additionally, cumulative incidences of relapse and NRM were not significantly different between European and Asian patients (Table 3). Patients with high PINK scores when compared to patients with low/intermediate PINK scores had higher NRM rates at 3 years with 17.5% (95% CI: 8.9–28.5%) vs. 3.4% (95% CI: 0.2–15.2%) (*P* = 0.07), which, however, did not significantly influence PFS- and OS-rates at 3 years [PFS: 50.9% (95% CI: 37.2–63.0%) vs. 61.4% (95% CI: 41.1–76.5%) (*P* = 0.51) and OS: 55.4% (95% CI: 41.2–67.5%) vs. 68.4% (95% CI: 47.9–82.2%) (*P* = 0.44)] (Table 3, Fig. 2B). ASPA–based treatment at any time before allo–HSCT was associated with a significantly higher relapse rate at 1 year [36.4% (95% CI: 26.7–46.1%) vs. 11.1% (95% CI: 3.4–23.9%) (*P* = 0.02)] and 3 years after allo–HSCT [40.4% (95% CI: 30.1–50.5%) vs. 17.1% (95% CI: 6.8–31.3%) (*P* = 0.02)] and, consequently, worse PFS rates [1-year PFS: 49.7% (95% CI: 39.2–59.3%) vs. 71.6% (95% CI: 53.6–83.6%) (*P* = 0.03); and 3-year PFS: 42.2% (95% CI: 31.8–52.2%) vs. 65.6% (95% CI: 47.4–78.9%) (*P* = 0.03)] (Table 3). Clinical characteristics of patients treated with ASPA- or non-ASPA-containing treatment prior to allo-HSCT, however, show important differences. The proportion of patients with high PINK scores was significantly higher in patients with ASPA-based treatment (*P* = 0.007) and the proportion of patients with more than 1 treatment line before allo-HSCT was higher in the ASPA-treated cohort as well (*P* = 0.002) (Supplementary Table 2). For pts with a shorter time interval (0–12 months) between diagnosis and allo-HSCT, we observed a larger proportion of advanced disease (stage III–IV) (*p* < 0.001) and high PINK scores (*p* = 0.007). Clinical characteristics by time from diagnosis to allo-HSCT are shown in Supplementary Table 3.

**Table 3:** Univariate analysis of post-transplantation outcomes for all patients.

Variable	Outcome	3-year probability [95% CI]		*P* value
Region		Europe	Asia	
	PFS	50.1% [39.2–60]	44.4% [28–59.6]	0.56
	OS	57.7% [46.8–67.1]	50.7% [33.4–65.6]	0.55
	RI	33.4% [23.7–43.3]	36.1% [20.7–51.7]	0.71
	NRM	16.5% [9.7–24.9]	19.4% [8.4–33.9]	0.71
PINK score at diagnosis		Low/intermediate	High	
	PFS	61.4% [41.1–76.5]	50.9% [37.2–63]	0.51
	OS	68.4% [47.9–82.2]	55.4% [41.2–67.5]	0.44
	RI	35.1% [18.1–52.7]	31.6% [19.9–44]	0.60
	NRM	3.4% [0.2–15.2]	17.5% [8.9–28.5]	0.07
Asparaginase before allo-HSCT		No	Yes	
	PFS	65.6% [47.4–78.9]	42.2% [31.8–52.2]	0.03
	OS	64.8% [46.4–78.3]	52.2% [41.4–61.9]	0.11
	RI	17.1% [6.8–31.3]	40.4% [30.1–50.5]	0.02
	NRM	17.3% [6.9–31.7]	17.4% [10.4–25.9]	1.00
PD–1/PD–L1 inhibitor before allo-HSCT		No	Yes	
	PFS	46.8% [36.5–56.4]	53.8% [24.8–76]	0.66
	OS	53.4% [42.9–62.9]	65.9% [31.5–86]	0.64
	RI	35.4% [25.9–45]	23.1% [4.9–48.9]	0.32
	NRM	17.8% [10.9–26.2]	23.1% [5.1–48.5]	0.57
Conditioning intensity		RIC	MAC	
	PFS	47.8% [34.6–59.8]	49.3% [36.6–60.7]	0.98
	OS	54.3% [40.8–66]	56.9% [44.1–67.8]	0.92
	RI	39.2% [26.7–51.4]	29.3% [18.7–40.7]	0.29
	NRM	13.1% [6–22.9]	21.4% [12.4–32.1]	0.19
Donor type		MRD/Unrelated	Haploidentical	
	PFS	50.5% [40.2–59.8]	39% [20.2–57.4]	0.45
	OS	55.8% [45.4–64.9]	52.6% [31.5–70]	0.71
	RI	32.6% [23.6–42]	40.7% [21–59.6]	0.61
	NRM	16.9% [10.3–24.9	20.3% [7.1–38.3]	0.67
Disease status at allo-HSCT		CR/PR	Non-CR/PR	
	PFS	52.1% [41.6–61.6]	37.0% [19.6–54.6]	0.08
	OS	60.6% [50.3–69.4]	38.8% [20.4–56.9]	0.06
	RI	29.7% [20.8–39]	51.9% [31.2–69]	0.02
	NRM	18.3% [11.3–26.5]	11.1% [2.7–26.4]	0.40

*CI* confidence interval, *PFS* progression-free survival, *OS* overall survival, *RI* cumulative incidence of relapse, *NRM* cumulative incidence of non-relapse mortality, *PINK* prognostic index for NK/T-cell lymphoma, *RIC* reduced-intensity conditioning, *MAC* myeloablative conditioning, *MRD* matched related donor, *CR/PR* complete response/partial response.

PD-1/PD-L1 inhibitors had been applied prior to HSCT to 13 patients; these patients did not show significant differences in terms of PFS or OS when compared to all other patients (Table 3). For PD-1/PD-L1 pre-treated patients remission status at transplantation was CR or PR in nine cases, relapse or progression in three cases, in one case remission status at transplantation was unavailable. Notably, we did not observe significant differences in these patients for acute GVHD grades II–IV at day 100 [11.1% (95% CI: 0.5–40.9%) vs. 19.0% (95% CI: 11.4–28.1%) (*P* = 0.56)] and all-grade chronic GVHD at 1 year [27.3% (95% CI: 5.5–55.9%) vs. 28.4% (95% CI: 19.5–37.9%) (*P* = 0.43)]. In the 5 patients who received post-transplantation PD-1/PD-L1 inhibitor treatment, we observed one case of acute GVHD grades II–IV at day 100 and 2 cases of chronic GVHD at 1 year after transplantation. With respect to the remission status at transplantation, we noted a higher relapse incidence for patients transplanted in relapse or with progressive disease compared to patients in CR or PR (*P* = 0.02) which was associated with a trend towards lower OS (*P* = 0.06) and PFS (*P* = 0.08) rates at 3 years (Table 3 and Fig. 2C). Regarding conditioning protocols, patients receiving RIC or MAC regimens had comparable outcomes in terms of PFS and OS (Table 3, Fig. 2D). While data showed a trend towards higher NRM after MAC [21.4% (95% CI: 12.4-32.1%) vs. 13.1% (95% CI: 6.0-22.9%, *P* = 0.19], the cumulative incidence of relapse did not show a significant difference [29.3% (95% CI: 18.7–40.7%) vs. 39.2% (95% CI: 26.7-51.4%, *P* = 0.29] (Table 3). Considering different donor types, no significant differences for any of the key outcome parameters were observed between patients with HLA-identical family donors, unrelated donors, and haplo-identical donors (Table 3). Of note, for transplants from haploidentical compared to HLA-matched related and unrelated donors, no significant differences in 3-year outcomes in terms of PFS (*P* = 0.45), OS (*P* = 0.71), relapse incidence (*P* = 0.61) or NRM (*P* = 0.67) were observed (Table 3).

### Univariate and multivariate regression analysis

Next, we built univariate and multivariate Cox regression models. As shown in Table 4, based on univariate analyses no significant differences with respect to PFS and OS were noted for sex, age at transplantation, stage at diagnosis, PINK score, number of prior treatment lines, PD-1/PD-L1 treatment before allo-HSCT, remission status at allo-HSCT, conditioning intensity, application of TBI, donor type or timing of allo-HSCT (1st CR/PR vs. 2nd or later CR/PR) (Table 4). In univariate comparisons, we noted a better PFS for patients who never had received ASPA as compared to ASPA-treated patients [HR (hazard ratio) = 1.91 (95% CI: 1.02–3.57); *P* = 0.04]. In the final multivariate model, however, the significant differences in terms of OS and PFS for patients with and without prior ASPA treatment were no longer present [OS: HR = 1.96 (95% CI: 0.71–5.46); *P* = 0.2 and PFS: HR = 2.52 (95% CI: 0.90–7.02); *P* = 0.08] (Table 4). The multivariate model showed that a shorter time interval (0–12 months) between diagnosis and allo-HSCT [HR = 2.12 (95% CI: 1.03–4.34); *P* = 0.04] significantly reduced PFS; pts transplanted not in remission (non-CR/PR) showed a trend for reduced PFS [HR = 2.20 (95% CI: 0.98–4.95); *P* = 0.056] (Table 4). This was largely driven by an increased risk of relapse in patients being transplanted in non-CR/PR [HR = 2.51 (95% CI: 1.00–6.34); *P* = 0.051].

**Table 4:** Univariate and multivariate Cox proportional hazards models of post-transplantation outcomes for all NKTCL patients.

	Overall Survival				Progression-free Survival			
	Univariate		Multivariate		Univariate		Multivariate	
Variable	HR [95% CI]	*P* value	HR [95% CI]	P-value	HR [95% CI]	*P* value	HR [95% CI]	*P* value
Sex: female vs. **male**	1.15 (0.67–1.97)	0.61			1.03 (0.61–1.74)	0.92		
Region: Europe vs. **East Asia**	0.84 (0.48–1.47)	0.55			0.85 (0.5–1.44)	0.55		
Disease Stage: III/IV vs. **I/II**	1.04 (0.59–1.84)	0.89			1.06 (0.61–1.83)	0.84		
PINK score at diagnosis: low/intermediate (0–1) vs. **high (>1)**	0.75 (0.37–1.54)	0.44	0.79 (0.36–1.76)	0.57	0.8 (0.4–1.57)	0.51	0.81 (0.39–1.70)	0.58
LDH at diagnosis: normal vs. **elevated**	0.58 (0.32–1.04)	0.07			0.74 (0.42–1.31)	0.3		
Age at allo-HSCT: 0–40 vs. **>40**	0.97 (0.58–1.62)	0.91			1.03 (0.63–1.68)	0.91		
Time to allo-HSCT: 0–12mo vs. **>12mo**	1.35 (0.81–2.26)	0.25	1.89 (0.88–4.02)	0.10	1.29 (0.79–2.1)	0.31	**2.12 (1.03–4.34)**	**0.04**
Status at allo-HSCT: non–CR **vs. CR**	1.27 (0.76–2.12)	0.36			1.17 (0.71–1.9)	0.54		
Status at allo–HSCT: non–CR/PR vs. **CR/PR**	1.73 (0.97–3.07)	0.06	2.14 (0.92–4.98)	0.08	1.64 (0.94–2.86)	0.08	**2.20 (0.98–4.95)**	**0.056**
Number of prior treatments: >1 vs. **1**	0.85 (0.46–1.58)	0.61			0.84 (0.46–1.52)	0.56		
PD-1/PD-L1 treatment before HSCT: yes vs. **no**	0.8 (0.32–2.02)	0.64			0.83 (0.35–1.93)	0.66		
Asparaginase before allo–HSCT: yes vs. **no**	1.57 (0.83–2.96)	0.16	1.96 (0.71–5.46)	0.20	**1.91 (1.02–3.57)**	**0.04**	2.52 (0.90–7.02)	0.08
Previous auto-HSCT: yes vs. **no**	0.52 (0.26–1.06)	0.07			0.64 (0.32–1.25)	0.19		
Conditioning intensity: RIC vs. **MAC**	1.03 (0.62–1.71)	0.9	1.39 (0.66–2.91)	0.39	1.02 (0.63–1.66)	0.94	1.25 (0.62–2.53)	0.53
TBI as part of conditioning: yes vs. **no**	1.43 (0.84–2.45)	0.19			1.40 (0.83–2.35)	0.21		
Donor type: Haploidentical vs. **MRD/Unrelated**	1.06 (0.57–1.97)	0.84			1.18 (0.66–2.1)	0.57		
CR/PR1 vs. **CR/PR2**	0.68 (0.35–1.31)	0.25			0.7 (0.37–1.31)	0.27		

References for Cox proportional hazards are highlighted in bold.

*HR* hazard ratio, *CI* confidence interval, *PINK* prognostic index for NK/T-cell lymphoma, *LDH* lactate dehydrogenase, *CR* complete response, *PR* partial response, *RIC* reduced-intensity conditioning, *MAC* myeloablative conditioning, *MRD* matched related donor, *CR/PR1* first CR/PR, *CR/PR2* second CR/PR, *HSCT* hematopoietic stem cell transplantation.

## Discussion

This study reports on the outcomes of Asian and European patients allografted for NKTCL. With the relatively high number of patients included, we provide reliable information on OS, PFS, NRM, and RI and identify prognostic factors having significant effects on survival. Outcomes of European and Asian patients were largely identical; the small remaining differences may be explained by differences in pretreatment and transplant modalities. Importantly, almost three-quarters of our patients had been treated with state-of-the-art ASPA-containing regimens prior to transplantation. The change from anthracycline- to ASPA-based regimens did not significantly affect PFS and OS after transplantation. Patients with different donor types including those with haplo-identical donors showed very similar PFS and OS rates confirming recent reports for lymphoma patients in general and for patients with T-cell lymphomas in particular [28–30]. Also, MAC and RIC regimens prior to transplantation gave comparable outcomes. The timing of allo-HSCT in first or later response did not influence survival rates. Although the proportion of patients treated with PD-1/PD-L1 inhibitors was rather small, we observed no significant differences in the frequency and severity of acute or chronic GVHD in patients who received PD-1/PD-L1 inhibitors before allo-HSCT. With only five patients treated, the role of PD-1/PD-L1 inhibitors given after transplantation for NK/ T-cell lymphoma remains unclear, although unusually early and severe GVHD was not observed.

With NKTCL being a very rare entity in Western countries, previous reports on allo-HSCT are mostly restricted to small retrospective studies, some of them clearly outdated in 2023. The CIBMTR study reported by Kanate et al. in 2018 including 82 patients transplanted between 2000 and 2014 is the largest study reported until now. The study included Caucasian/ other (*n* = 66) and a few Asian (*n* = 16) patients, also without reporting significant differences in outcome [21]. However, the 3-year PFS and OS were only 28% and 34%, respectively. The risk factors identified in this analysis, namely the time interval between diagnosis and transplantation as well as the proportion of patients transplanted in CR/PR, do not appear different when compared to the analysis by Kanate et al. [21]. We have no convincing explanation at hand why for patients in our study PFS and OS are almost twice as high. However, given the fact that patients were allografted in earlier years in the CIBMTR analysis, differences in patient characteristics, pretreatment, donor selection, conditioning, and GVHD prophylaxis may all play a role.

Data from smaller Asian studies before the broader application of ASPA-containing regimens had reported 2-year PFS and OS rates of 34% and 40%, respectively [16, 31]. More recent analyses from Asia and the Western hemisphere involving limited patient numbers pretreated with ASPA-containing regimens in ~70% of the cases report PFS and OS rates between 26–51% and 52–57% which seem more comparable to our results [17, 20, 32]. Relapse was the main cause of treatment failure and death after allo-HSCT in most studies on NKTCL [17, 20, 21, 31, 32]. These findings, together with the fact that most relapses occurred within 1–2 years after transplantation are well in line with our data. In contrast to previous reports, we found an NRM rate of only 15% at 1 year which compares favorably to previous studies and might be explained by better donor selection, GVHD prophylaxis, supportive care, and the development of RIC regimens during the past decades [17, 20, 21, 31, 32]. The low NRM might in part explain the better PFS and OS observed in our study.

In terms of ASPA treatment before allo-HSCT, we did not observe significant differences for any major outcome parameter in the final multivariate model after adjusting for risk factors found in the univariate analyses. Tse et al. reported significantly better event-free survival for patients with ASPA-based treatment using the SMILE protocol (dexamethasone, methotrexate, ifosfamide, L-asparaginase, and etoposide) prior to allo-HSCT [20] whereas other studies did not observe significant differences in outcomes with prior ASPA [21, 32]. Given these, in part contradictory results, we speculate that the use of ASPA-containing therapy may bring more patients to allo-HSCT but seems to have a limited if any impact on transplant results in themselves.

As shown in previous reports of smaller cohorts and for other lymphoma entities, we confirm that achieving a CR or PR prior to allo-HSCT improves the post-transplantation outcome [20, 21, 32, 33]. It is noteworthy that patients with haplo-identical donors had outcomes well compared to all other donor types. This seems to be a consequence of altered GVHD prophylaxis and is in line with previous reports [34]. Our data support a more permissive use of haplo-identical donors to allow for timely transplantation of NKTCL patients whenever related or (matched) unrelated donors are not readily available.

In terms of conditioning, our findings are consistent with other reports indicating that RIC and MAC protocols achieve comparable outcomes. In NKTCL, significant differences between MAC or RIC for any major outcome parameter were not previously reported [16, 17, 21, 31, 32].

Immune checkpoint inhibitors targeting the PD-1-/PD-L1 axis have been shown to be effective in relapsed or refractory NKTCL in several uncontrolled and mainly retrospective studies [14, 15]. The debate on how PD-1/PD-L1 antibodies perform prior and after allo-HSCT for different types of lymphoma is ongoing. While early reports suggested aggravated GVHD and higher NRM, more recent analyses did not confirm such findings [35–38]. Other reports on patients with NKTCL undergoing allo-HSCT after PD-1/PD-L1 antibody treatment are not available. Although our experience is restricted to 13 patients only, we did not observe that administration of PD-1/PD-L1 antibodies before allo-HSCT caused more or more severe GVHD and increases in NRM. We rather believe that the administration of PD-1/PD-L1 antibodies before transplantation might bring more patients to allo-HSCT sparing these patients the side effects of aggressive chemotherapy. Whether PD-1/PD-L1 antibodies can safely be administered after allo-HSCT to prevent disease relapse in high-risk patients warrants further research in larger groups of patients; early reports in patients with Hodgkin lymphoma were not encouraging [35, 39].

Our study has limitations inherent to any retrospective analysis. Most importantly, we cannot know how many patients had been scheduled for allo-HSCT but were unable to receive this treatment because salvage therapy was unsuccessful, and patients were no longer considered candidates for transplantation. The availability of PD-1/ PD-L1 antibodies and other targeted therapies prior to allo-HSCT as well as the broader use of haplo-identical transplantation might, however, contribute to closing the gap between patients needing and those proceeding to allo-HSCT. Additionally, we cannot exclude that auto-HSCT may achieve results comparable to allo-HSCT in selected patients achieving CR/PR prior to transplantation avoiding the higher NRM after allo-HSCT. A recent analysis of French data showed results for 19 allo- and 46 auto-grafted patients, but no comparison between allo- and auto-HSCT was performed [32]. Therefore, representative and comparative outcome results for allo- and auto-HSCT in NKTCL patients remain a question for future analyses.

In conclusion, we present reliable outcome data for a large international cohort confirming that allo-HSCT is an effective treatment approach for NKTCL patients achieving long-term survival in more than half of the cases. As these results were obtained with the majority of patients having been treated with state-of-the-art ASPA-containing therapy prior to transplantation, allo-HSCT should be considered the preferred option in medically fit patients with relapsed or refractory NKTCL. With the current lack of new drugs and cellular therapies that might improve the outcomes of patients with relapsed or refractory NKTCL further research to reduce relapse incidence as well as non-relapse mortality after allo-HSCT for NKTCL is highly warranted.

## Supplementary information

SUPPLEMENTAL MATERIAL

## Data Availability

The datasets presented in the study are included in the article / Supplementary information. Further inquiries can be directed to the corresponding author.
