# Supplementary material for: Allogeneic hematopoietic stem cell transplantation for NK/T-cell lymphoma: an international collaborative analysis
Source: Leukemia. 2023 May 8;37(7):1511–20. doi: 10.1038/s41375-023-01924-x (PMC10166457; doi:10.1038/s41375-023-01924-x)
Supplement: Supplementary file 1 — SUPPLEMENTAL MATERIAL [file 41375_2023_1924_MOESM1_ESM.docx]

# Supplementary Information

**Allogeneic Hematopoietic Stem Cell Transplantation for NK/T-cell Lymphoma: An International Collaborative Analysis**

Philipp Berning*^1*^*, Norbert Schmitz*^1*^*, Maud Ngoya*^2^*, Hervé Finel*^2^*, Ariane Boumendil*^2^*, Fengrong Wang*^3^*, Xiao-Jun Huang*^3^*, Olivier Hermine*^4^*, Laure Philippe*^4^*, Lucile Couronné*^5^*, Arnaud Jaccard*^6^*, Daihong Liu*^7^*, Depei Wu*^8^*, Hans Christian Reinhardt*^9^*, Yves Chalandon*^10^*, Eva Wagner-Drouet*^11^* , Mi Kwon*^12^,* Xi Zhang*^13^*, Ben Carpenter*^14^*, Ibrahim Yakoub-Agha*^15^,* Gerald Wulf*^16^,* Javier López-Jiménez*^17^*, Jaime Sanz*^18^,* Hélène Labussière-Wallet*^19^*, Avichai Shimoni*^20^*, Peter Dreger*^21^*, Anna Sureda*^22^*, Won Seog Kim*^23#^* and Bertram Glass*^24#^*

*1 Department of Hematology and Oncology, University Hospital Muenster, Muenster, Germany
2 European Society for Blood and Marrow Transplantation, Hôpital St. Antoine, Paris, France*

*3 Beijing University, Institute of Hematology, National Clinical Research Center for Hematologic Disease, Beijing, China*

*4 Department of Hematology, Hôpital Necker, Assistance Publique Hôpitaux de Paris, University Paris Descartes, Paris, France*

*5 Laboratory of Onco-Hematology, Necker Children's Hospital, Assistance Publique-Hôpitaux de Paris (APHP), Université Paris Cité, Paris, France*

*6 Department of Hematology, CHU Dupuytren, Limoges, France*

*7 General Hospital of People's Liberation Army of China, Beijing, China*

*8 National Clinical Research Center for Hematologic Diseases, Jiangsu Institute of Hematology, The First Affiliated Hospital of Soochow University, Soochow, China*

*9 Department of Hematology and Stem Cell Transplantation, University Hospital Essen, University Duisburg-Essen, Essen, Germany*

*10 Division of Hematology, Hôpitaux Universitaires de Genève and Faculty of Medicine, University of Geneva, Geneva, Switzerland*

*11 Department of Medicine III, Johannes Gutenberg-University Mainz, Mainz, Germany*

*12 Department of Hematology, Gregorio Marañón General University Hospital, Institute of Health Research Gregorio Marañón, Madrid, Spain*

*13 Medical center of Hematology, Xinqiao Hospital, Army Medical University, Chongqing, China*

*14 Department of Haematology, University College London Hospitals, London, United Kingdom*

*15 CHU de Lille, INSERM U1286, Infinite, Université de Lille, Lille, France*

*16 Department of Hematology/Oncology, Georg-August-University Göttingen, Göttingen, Germany*

*17 Hematology Department, Hospital Universitario Ramón y Cajal, Madrid, Spain*

*18 Department of Hematology, Hospital Universitario y Politécnico La Fe, Valencia, Spain*

*19 Centre Hospitalier Lyon Sud, Pavillon Marcel Bérard, Service Hematologie, Lyon, France*

*20 Division of Hematology and Bone Marrow Transplantation, Chaim Sheba Medical Center, Tel-Hashomer, Israel*

*21 Department of Medicine V, University of Heidelberg, Heidelberg, Germany*

*22 Hematology Department, Institut Català d'Oncologia Hospitalet, IDIBELL, Universitat de Barcelona, Barcelona, Spain*

*23 Samsung Medical Center, Sungkyunkwan University School of Medicine, Seoul, Korea*

*24 Department of Hematology and Stem Cell Transplantation, Helios Clinic, Berlin-Buch, Germany*

^*^ Co-first authors; ^#^ Co-senior authors

**^†^ Corresponding authors:** Philipp Berning, Department of Hematology and Oncology, University Hospital Muenster, Albert-Schweitzer-Campus 1 A1, 48149 Muenster, Germany; email: philipp.berning@ukmuenster.de

Presented in abstract form at the 64th annual meeting of the American Society of Hematology in New Orleans, LA, December 10-13, 2022. P.B. received a 2022 ASH Abstract Achievement Award**.**

**Supplemental Table 1 Cooperating centers listed by patient numbers.**

| **Center** | **Patient numbers** |
| --- | --- |
| Peking University People's Hospital, Beijing, China | 12 |
| Samsung Medical Center Seoul, South Korea | 8 |
| PLA General Hospital, Beijing, China | 7 |
| First Affiliated Hospital of Soochow University, Suzhou, China | 5 |
| University Hospital Essen, Germany | 4 |
| Hôpitaux Universitaires Geneva, Switzerland | 4 |
| University Medical Center Mainz, Germany | 4 |
| Hospital Gregorio Marañón Madrid, Spain | 4 |
| Xinqiao Hospital, Army Medical University, Chongqing, China | 4 |
| University College London Hospital London, United Kingdom | 3 |
| Hôpital Huriez Lille, France | 3 |
| Universitaetsklinikum Gottingen, Germany | 3 |
| Hospital Ramón y Cajal Madrid, Spain | 3 |
| University Hospital La Fe Valencia, Spain | 3 |
| Centre Hospitalier Lyon Sud Lyon, France | 3 |
| Chaim Sheba Medical Center Tel-Hashomer, Israel | 3 |
| Hopital St. Louis, Paris, France | 2 |
| Medical School Hannover, Germany | 2 |
| Fundación Jiménez Díaz Madrid, Spain | 2 |
| Singapore General Hospital, Singapore | 2 |
| Klinikum Rechts der Isar, Munich, Germany | 2 |
| Policlinico G.B. Rossi, Verona, Italy | 2 |
| Institut Gustave Roussy, Villejuif, France | 2 |
| Universitaetsklinikum Wuerzburg, Germany | 2 |
| CHU Sart-Tilman, Liege, Belgium | 2 |
| University Regensburg, Germany | 2 |
| Charité Universitätsmedizin, Berlin, Germany | 2 |
| Robert-Bosch-Krankenhaus, Stuttgart, Germany | 1 |
| Hôpital Necker, Paris, France | 1 |
| University Hospital, Basel, Switzerland | 1 |
| Karolinska University Hospital, Stockholm, Sweden | 1 |
| Hospital Clinic, Barcelona, Spain | 1 |
| Royal Marsden Hospital, London, United Kingdom | 1 |
| Medizinische Klinik, Tübingen, Germany | 1 |
| Cliniques Universitaires St. Luc, Brussels, Belgium | 1 |
| Hosp. Reina Sofia, Cordoba, Spain | 1 |
| Erasmus MC Cancer Institute, Rotterdam, Netherlands | 1 |
| Ospedale Civile, Pescara, Italy | 1 |
| Institut de Cancerologie Lucien Neuwirth, Saint Etienne, France | 1 |
| CHU, Nantes, France | 1 |
| Hospital Santa Creu i Sant Pau, Barcelona, Spain | 1 |
| Hopital de la Pitié-Salpêtrière, Paris, France | 1 |
| CHU, Grenoble, France | 1 |
| Rambam Medical Center, Haifa, Israel | 1 |
| Oncology Center, Gliwice, Poland | 1 |
| Klinikum Grosshadern, Munich, Germany | 1 |
| HELIOS Klinikum, Berlin-Buch, Germany | 1 |
| University of Heidelberg, Heidelberg, Germany | 1 |
| University of Cologne, Germany | 1 |
| Hospital Univ. Virgen de las Nieves, Granada, Spain | 1 |
| Hospital Regional de Málaga, Spain | 1 |
| Complejo Hospitalario de Navarra, Pamplona, Spain | 1 |
| Royal Infirmary, Manchester, United Kingdom | 1 |
| Klinikum Bremen-Mitte, Germany | 1 |
| University Hospital Eppendorf, Hamburg, Germany | 1 |
| Istituto Nazionale dei Tumori, Milano, Italy | 1 |
| CHRU, Angers, France | 1 |
| C.H.R.U de Brest, France | 1 |
| Nouvel Hopital Civil, Strasbourg, France | 1 |
| University of Münster, Münster, Germany | 1 |
| Nottingham University, Nottingham, United Kingdom | 1 |
| Charles University Hospital, Pilsen, Czech Republic | 1 |
| University Hospital, Umeå, Sweden | 1 |
| GKT School of Medicine, London, United Kingdom | 1 |
| Hôpital Saint Antoine, Paris, France | 1 |
| Universitaetsklinikum, Dresden, Germany | 1 |
| University of Freiburg, Freiburg, Germany | 1 |
| Ospedale San Raffaele, Milano, Italy | 1 |
| Florence Nightingale Sisli Hospital, Istanbul, Turkey | 1 |

**Supplemental Table 2.** **Baseline characteristics of patients with Asparaginase-containing treatments prior to allo-HSCT**

| **Variable** | **No ASPA prior to allo-HSCT**  **N = 35 (%)** | **ASPA prior to allo-HSCT**  **N = 100 (%)** | **P-Value** |
| --- | --- | --- | --- |
| Male Sex | 21 (60.0) | 71 (71.0) | 0.229 |
| Age at diagnosis, median (years) | 44.3 (23.9-62.2) | 40.9 (15.8-66.2) | 0.365 |
| Age at transplantation, median (years) | 45.5 (25.4-62.8) | 42.6 (18.3-67.7) | 0.388 |
| Diagnosis – allo-HSCT, median (months) | 11.0 (1.6-89.1) | 11.2 (3.2-257) | 0.859 |
| Karnofsky performance score at allo-HSCT |  |  | 0.665 |
| 80-100% | 23 (65.7) | 62 (62.0) |  |
| <80% | 2 (5.7) | 4 (4.0) |  |
| Unknown | 10 (28.6) | 34 (34.0) |  |
| Region |  |  | 0.093 |
| Europe | 29 (82.9) | 68 (68.0) |  |
| Asia | 6 (17.1) | 32 (32.0) |  |
| Ann-Arbor stage at diagnosis |  |  | 0.211 |
| Localized (I-II) | 12 (34.3) | 28 (28.0) |  |
| Advanced (III-IV) | 16 (45.7) | 67 (67.0) |  |
| Unknown | 7 (20.0) | 5 (5.0) |  |
| PINK score |  |  | 0.007 |
| Low/Intermediate | 3 (8.6) | 26 (26.0) |  |
| High | 13 (37.1) | 47 (47.0) |  |
| Unknown | 19 (54.3) | 27 (27.0) |  |
| First-line therapy |  |  | <0.001 |
| Anthracycline-based | 20 (57.1) | 16 (16.0) |  |
| Asparaginase-based | 0 (0) | 68 (68.0) |  |
| DeVIC or VIPD | 1 (2.9) | 7 (7.0) |  |
| Gemcitabine-based | 1 (2.9) | 0 (0) |  |
| Other | 8 (22.8) | 6 (6.0) |  |
| Unknown | 5 (14.38) | 3 (3.0) |  |
| Radiotherapy in first-line | 10 (28.6) | 34 (34.0) | 0.849 |
| Prior autologous transplantation | 10 (28.6) | 18 (18.0) | 0.184 |
| Number of prior therapies |  |  | 0.002 |
| 1 | 6 (17.1) | 21 (21.0) |  |
| >1 | 24 (69.4) | 79 (78.0) |  |
| Unknown | 5 (14.3) | 0 |  |
| PD-1/PD-L1 inhibitor treatment |  |  | 0.123 |
| No PD-1/PD-L1 inhibitor | 23 (65.7) | 73 (73.0) |  |
| After HSCT | 1 (2.9) | 4 (4.0) |  |
| Before HSCT | 0 (0) | 13 (13.0) |  |
| Unknown | 11 (31.4) | 10 (10.0) |  |
| Status at transplantation |  |  |  |
| CR | 15 (42.9) | 56 (56.0) | 0.612 |
| PR | 11 (31.4) | 25 (25.0) |  |
| SD | 1 (2.9) | 2 (2.0) |  |
| PD | 8 (22.9) | 16 (16.0) |  |
| Unknown | 0 | 1 (1.0) |  |
| Donor Type |  |  | 0.562 |
| Matched related donor | 14 (40.0) | 34 (34.0) |  |
| Unrelated donor | 17 (48.6) | 42 (42.0) |  |
| Mismatched related donor | 4 (11.4) | 23 (23.0) |  |
| Haploidentical donor | 4 (11.4) | 23 (23.0) |  |
| Other | 0 | 1 (1.0) |  |
| Stem cell source |  |  | 1.0 |
| Bone marrow | 3 (8.6) | 9 (9.0) |  |
| Peripheral blood | 32 (91.4) | 91 (91.0) |  |
| Conditioning regimen |  |  | 0.358 |
| RIC | 15 (41.7) | 49 (49.0) |  |
| MAC | 21 (58.3) | 51 (51.0) |  |
| TBI as part of conditioning | 8 (22.9) | 27 (27.0) | 0.630 |
| >6 Gy | 2 (5.7) | 6 (6.0) |  |
| ≤ 6 Gy | 4 (11.4) | 13 (13.0) |  |
| TBI, but dose unknown | 2 (5.7) | 8 (8.0) |  |
| No TBI | 27 (77.1) | 73 (73.0) |  |
| In vivo T-cell depletion |  |  | 0.954 |
| ATG | 13 (37.1) | 37 (37.0) |  |
| Alemtuzumab | 3 (8.6) | 7 (7.0) |  |
| No T-cell depletion | 19 (54.3) | 56 (56.0) |  |
| PTCY |  |  | 0.630 |
| Yes | 8 (22.9) | 27 (27.0) |  |
| No | 27 (77.1) | 73 (73.0) |  |
| GVHD prophylaxis |  |  |  |
| Cyclosporin A alone | 4 (11.4) | 10 (10.0) |  |
| Cyclosporin A + MTX | 12 (34.3) | 21 (21.0) |  |
| Cyclosporin A + MMF | 5 (14.3) | 13 (13.0) |  |
| Cyclosporin A + MMF + MTX | 6 (17.1) | 21 (21.0) |  |
| Tacrolimus + MTX | 1 (2.9) | 5 (5.0) |  |
| Tacrolimus + MMF | 2 (5.7) | 4 (4.0) |  |
| Other | 5 (14.3) | 25 (25.0) |  |
| Unknown | 0 | 1 (1.0) |  |
| Abbreviations: PINK=prognostic index for NK/T-cell lymphoma; DeVIC=dexamethasone, etoposide, ifosfamide, carboplatin; VIPD=etoposide, ifosfamide, cisplatin, dexamethasone; CR=complete response; PR=partial response; SD=stable disease; PD=progressive disease; RIC=reduced-intensity conditioning; MAC=myeloablative conditioning; TBI=total body irradiation; ATG=anti-thymocyte globulin; PTCY=post-transplant cyclophosphamide; GVHD=graft-versus-host disease; MTX=methotrexate; MMF=mycophenolate mofetil. | | | |

**Supplemental Table 3.** **Baseline characteristics by the time between diagnosis and allo-HSCT**

| **Variable** | **Time diagnosis**  – **allo-HSCT** | | **P-Value** |
| --- | --- | --- | --- |
|  | **>12 months**  **N = 64 (%)** | **0-12 months**  **N = 71 (%)** |  |
| Male Sex | 43 (67.2) | 49 (69.0) | 0.820 |
| Age at diagnosis, median (years) | 42.0 (15.8-66.2) | 41.8 (18.9-65.4) | 0.951 |
| Age at transplantation, median (years) | 45.4 (18.3-67.7) | 42.1 (19.7-66.3) | 0.331 |
| Karnofsky performance score at allo-HSCT |  |  | 0.091 |
| 80-100% | 48 (75.0) | 37 (52.1) |  |
| <80% | 1 (1.6) | 5 (7.0) |  |
| Unknown | 15 (23.4) | 29 (40.9) |  |
| Region |  |  | 0.007 |
| Europe | 53 (82.8) | 44 (62.0) |  |
| Asia | 11 (17.2) | 27 (38.0) |  |
| Ann-Arbor stage at diagnosis |  |  | <0.001 |
| Localized (I-II) | 29 (45.3) | 11 (15.5) |  |
| Advanced (III-IV) | 26 (40.6) | 57 (80.3) |  |
| Unknown | 9 (14.1) | 3 (4.2) |  |
| PINK score |  |  | 0.007 |
| Low/Intermediate | 18 (28.1) | 11 (15.5) |  |
| High | 19 (29.7) | 41 (57.7) |  |
| Unknown | 27 (42.2) | 19 (26.8) |  |
| First-line therapy |  |  | 0.115 |
| Anthracycline-based | 23 (35.9) | 13 (18.3) |  |
| Asparaginase-based | 28 (43.8) | 40 (56.3) |  |
| DeVIC or VIPD | 3 (4.7) | 5 (7.0) |  |
| Gemcitabine-based | 0 (0) | 1 (1.4) |  |
| Other | 5 (7.8) | 9 (12.7) |  |
| Unknown | 5 (7.8) | 3 (4.2) |  |
| Radiotherapy in first-line | 34 (54.4) | 10 (14.1) | <0.001 |
| Prior autologous transplantation | 27 (42.2) | 1 (1.4) | <0.001 |
| Number of prior therapies |  |  | <0.001 |
| 1 | 1 (1.6) | 26 (36.6) |  |
| >1 | 62 (96.8) | 41 (57.7) |  |
| Unknown | 1 (1.6) | 4 (5.7) |  |
| PD-1/PD-L1 inhibitor treatment |  |  | 0.852 |
| No PD-1/PD-L1 inhibitor | 43 (67.2) | 53 (74.6) |  |
| After HSCT | 2 (3.1) | 3 (4.2) |  |
| Before HSCT | 7 (10.9) | 6 (8.5) |  |
| Unknown | 12 (18.8) | 9 (12.7) |  |
| Status at transplantation |  |  | 0.403 |
| CR | 33 (51.6) | 37 (53.5) |  |
| PR | 16 (25.0) | 20 (28.2) |  |
| SD | 3 (2.2) | 0 (0) |  |
| PD | 12 (18.8) | 12 (16.9) |  |
| Unknown | 0 | 1 (1.4) |  |
| Donor Type |  |  | 0.105 |
| Matched related donor | 24 (37.5) | 24 (33.8) |  |
| Unrelated donor | 30 (46.9) | 29 (40.8) |  |
| Mismatched related donor | 10 (15.6) | 18 (25.4) |  |
| Stem cell source |  |  | 0.850 |
| Bone marrow | 6 (9.4) | 6 (8.5) |  |
| Peripheral blood | 58 (90.6) | 65 (91.5) |  |
| Conditioning regimen |  |  | 0.014 |
| RIC | 37 (57.8) | 26 (36.6) |  |
| MAC | 27 (42.2) | 45 (63.4) |  |
| TBI as part of conditioning |  |  | 0.603 |
| >6 Gy | 6 (9.4) | 11 (15.5) |  |
| ≤ 6 Gy | 5 (7.8) | 3 (4.2) |  |
| TBI, but dose unknown | 4 (6.2) | 6 (8.5) |  |
| No TBI | 49 (76.6) | 51 (71.8) |  |
| In vivo T-cell depletion |  |  | 0.321 |
| ATG | 23 (35.9) | 27 (38.0) |  |
| Alemtuzumab | 7 (10.9) | 3 (4.2) |  |
| No T-cell depletion | 34 (53.1) | 41 (57.7) |  |
| PTCY |  |  | 0.461 |
| Yes | 16 (25.0) | 14 (19.7) |  |
| No | 48 (75.0) | 57 (80.3) |  |
| Abbreviations: PINK=prognostic index for NK/T-cell lymphoma; DeVIC=dexamethasone, etoposide, ifosfamide, carboplatin; VIPD=etoposide, ifosfamide, cisplatin, dexamethasone; CR=complete response; PR=partial response; SD=stable disease; PD=progressive disease; RIC=reduced-intensity conditioning; MAC=myeloablative conditioning; TBI=total body irradiation; ATG=anti-thymocyte globulin; PTCY=post-transplant cyclophosphamide. | | | |
